# Evaluating the effect of SARS-CoV-2 spike mutations with a linear doubly robust learner

**DOI:** 10.3389/fcimb.2023.1161445

**Published:** 2023-04-19

**Authors:** Xin Wang, Mingda Hu, Bo Liu, Huifang Xu, Yuan Jin, Boqian Wang, Yunxiang Zhao, Jun Wu, Junjie Yue, Hongguang Ren

**Affiliations:** Beijing Institute of Biotechnology, State Key Laboratory of Pathogen and Biosecurity, Beijing, China

**Keywords:** SARS-CoV-2, mutation, fitness, causal inference, basic reproduction number (R0)

## Abstract

Driven by various mutations on the viral Spike protein, diverse variants of SARS-CoV-2 have emerged and prevailed repeatedly, significantly prolonging the pandemic. This phenomenon necessitates the identification of key Spike mutations for fitness enhancement. To address the need, this manuscript formulates a well-defined framework of causal inference methods for evaluating and identifying key Spike mutations to the viral fitness of SARS-CoV-2. In the context of large-scale genomes of SARS-CoV-2, it estimates the statistical contribution of mutations to viral fitness across lineages and therefore identifies important mutations. Further, identified key mutations are validated by computational methods to possess functional effects, including Spike stability, receptor-binding affinity, and potential for immune escape. Based on the effect score of each mutation, individual key fitness-enhancing mutations such as D614G and T478K are identified and studied. From individual mutations to protein domains, this paper recognizes key protein regions on the Spike protein, including the receptor-binding domain and the N-terminal domain. This research even makes further efforts to investigate viral fitness *via* mutational effect scores, allowing us to compute the fitness score of different SARS-CoV-2 strains and predict their transmission capacity based solely on their viral sequence. This prediction of viral fitness has been validated using BA.2.12.1, which is not used for regression training but well fits the prediction. To the best of our knowledge, this is the first research to apply causal inference models to mutational analysis on large-scale genomes of SARS-CoV-2. Our findings produce innovative and systematic insights into SARS-CoV-2 and promotes functional studies of its key mutations, serving as reliable guidance about mutations of interest.

## 1 Introduction

As of Jan 2023, the coronavirus disease 2019 (COVID-19) pandemic, caused by severe acute respiratory syndrome coronavirus 2 (SARS-CoV-2) (Zhou et al., 2020), has been ongoing for more than three years, resulting in over 754 million infections and 6.8 million deaths worldwide (https://covid19.who.int/). As a paramount characteristic of SARS-CoV-2, diverse variants have emerged and prevailed repeatedly, driven by numerous mutations, particularly on the viral Spike protein (Harvey et al., 2021; Kang et al., 2021). These emerging variants of SARS-CoV-2 have substantially prolonged the pandemic by causing repeated epidemics, posing a continuing threat to public health across the world (Obermeyer et al., 2022).

During the pandemic, the Spike protein of SARS-CoV-2 has attracted particular attention because it functionally mediates viral entry into host cells (Shang et al., 2020), and is the target of antibody-mediated immunity (Gaebler et al., 2021; McCallum et al., 2021; Shah et al., 2021). Meanwhile, various mutations have accumulated in the Spike protein, including the receptor-binding domain (RBD, amino acid position 319-541), which may enhance viral fitness and give rise to new variants (Harvey et al., 2021). For instance, the D614G mutation can increase viral infectivity (Hou et al., 2020; Korber et al., 2020; Yurkovetskiy et al., 2020) and has been found in almost all the following VoCs (Variant of Concern). Therefore, it is crucial to identify key Spike mutations that likely elevate viral fitness for further research on SARS-CoV-2.

Up until now, millions of genome sequences of SARS-CoV-2 have been submitted and shared globally (Shu and McCauley, 2017), making computational analysis on viral mutations feasible. As a novel computing method, causal inference model enjoys broad prospects for applications (Pearl, 2009; Yao et al., 2021). It produces an unbiased estimation of the effect of a given intervention with confounding factors (Pearl, 2009; Guo et al., 2020; Yao et al., 2021). Those models are particularly applicable to mutational analysis on SARS-CoV-2, in which mutations act as confounding factors to each other. With the benefits of causal inference models, Spike mutations can be evaluated according to the statistical contribution to viral fitness, in the context of large-scale genomes of SARS-CoV-2. Subsequently, key fitness-enhancing mutations can be identified and distinguished from numerous mutations, validated for their mutational effects by various methods, and further applied to downstream analysis.

**Figure 1:**
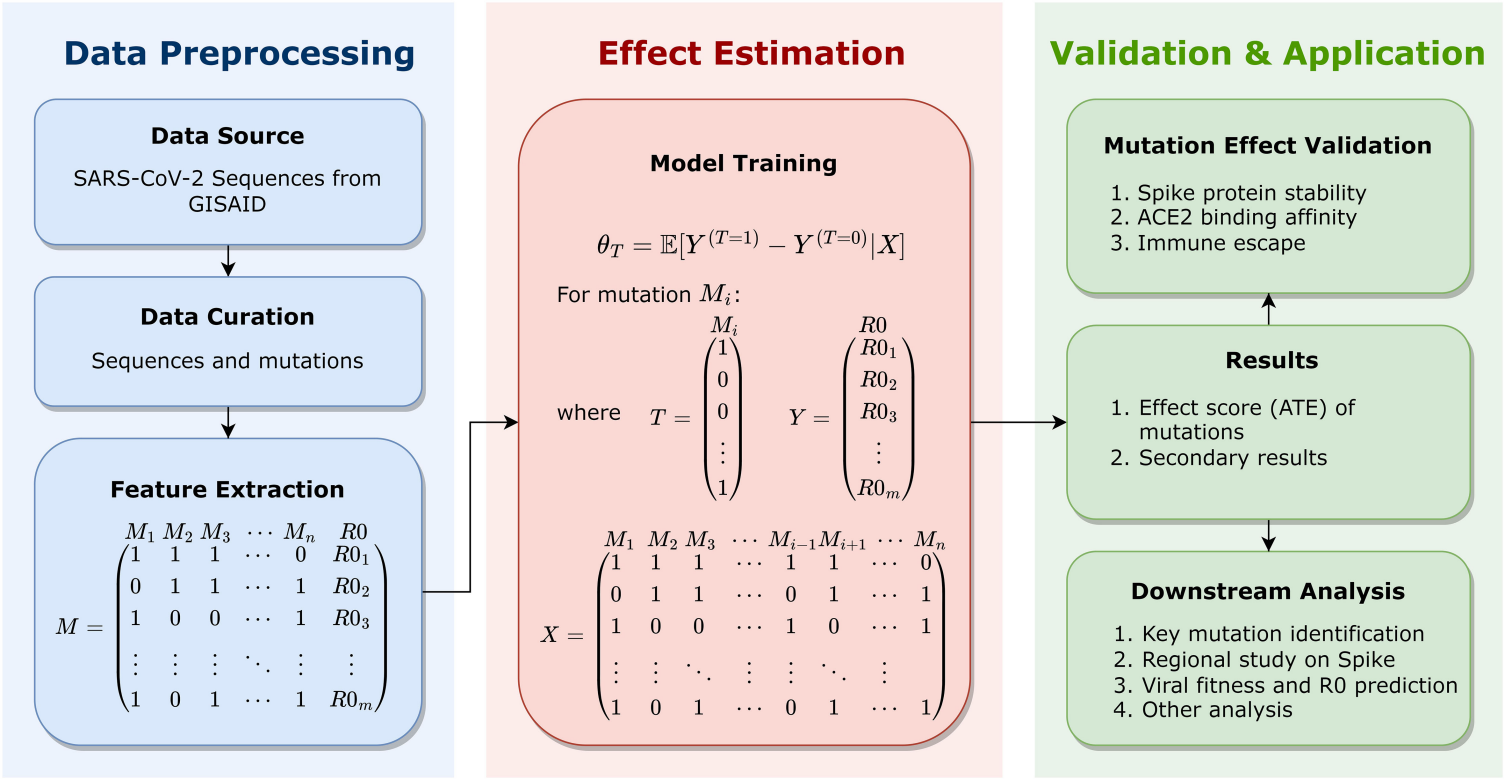
Schematic representation for the framework of this study, including the Data Preprocessing, the Effect Estimation, and the Validation and Application. Genome sequences of SARS-CoV-2 are retrieved and integrated for data curation and feature extraction, which are presented as FASTA files of bases (A, C, G, and T). In data curation, Spike sequences are aligned to the reference sequence for mutation detection; in feature extraction, each sequence is mapped into a mutation combination (as a boolean vector representing the existence of mutations in each sequence) with the corresponding R0, as a row vector in the feature matrix. For each mutation, the causal inference model is utilized to estimate its average treatment effect (ATE) on the outcome R0, with other mutations serving as observable covariates. Estimated ATE serves as the effect score of each mutation. Identified key mutations are validated and interpreted by computing methods for detailed mutational influences, including the Spike protein stability, the human angiotensin-converting enzyme 2 (ACE2) binding affinity, and immune escape. Effect scores can support downstream analysis, including key mutation identification, region study of the Spike protein, viral fitness and R0 prediction of strains, etc.

This manuscript formulates a well-defined framework that utilizes causal inference models to estimate the statistical contribution of Spike mutations to viral fitness across lineages. To the best of our knowledge, this is the first research to apply causal inference models to mutational analysis on large-scale genomes of SARS-CoV-2. This work, as schematically depicted in **Figure 1** and described in detail in the Methodology section, includes the Data Preprocessing, the Effect Estimation, the Validation and Application, etc. In the Data Preprocessing stage, 7.7 million high-quality SARS-CoV-2 complete genome sequences as of May 11, 2022 are retrieved from GISAID website (Shu and McCauley, 2017), aligned for Spike amino acid mutations, and mapped into mutation combinations with the corresponding basic reproduction number (R0), as row vectors in the feature matrix. In the Effect Estimation stage, the causal inference model is utilized for an unbiased estimation of the average treatment effect (ATE) of each mutation on the outcome R0. The estimated ATE serves as the effect score of mutations, based on which important mutations can be identified. Further, identified key mutations are validated by computational methods that assess their mutational influences, including the Spike protein stability, the host cell-surface receptor (the human angiotensin-converting enzyme 2, ACE2) binding affinity, and the potential for immune escape. Therefore, key mutations can be identified, validated, and also interpreted in details. Based on effect scores as the quantitative assessment of mutations, important mutations can be recognized and investigated. From individual mutations to protein regions, this paper recognizes key protein regions on the Spike protein. This research even makes further efforts to investigate viral fitness *via* mutational effect scores. By the effect score of mutations, the fitness of SARS-CoV-2 variants is estimated, which can be utilized for viral fitness prediction by a trained regression. This regression is validated by BA.2.12.1, which is not used for regression training but well fits the prediction. Using this method, the transmission capacity of any new variant can be predicted solely based on the viral sequence. Moreover, secondary results of causal inference models can likewise assist further analysis, which may reveal potential interactions between mutations. This research produces innovative and systematic insights into SARS-CoV-2 and promotes functional studies of its key mutations, which may contribute to the evolutionary characterization of SARS-CoV-2 and the development of Spike-targeted medicines and vaccines against SARS-CoV-2.

## 2 Results

This research aims to estimate the statistical contribution of Spike mutations to the viral fitness of SARS-CoV-2 and identify important mutations, as depicted in **Figure 1**. Generally, the Results section is comprised of four parts. Firstly, this section presents the preprocessing, core estimation on mutations, and validations of identified key mutations. Secondly, important mutations on the Spike protein are explored, particularly from the perspective of structural conformations. In the next subsection, a regional study on the Spike protein is conducted to identify critical mutational regions from individual mutations. Finally, this section investigates the results of viral fitness and R0 prediction *via* effect scores.

### 2.1 Effect score and validation of mutations

In this subsection, we firstly present the estimation on mutations and the identification of significant mutations. Then, this subsection validates mutational effects through computational methods and supportive references to strengthen the effectiveness of this research. Additionally, this research conducts a biological experimental study on mutant RBD based on identified key mutations.

#### 2.1.1 Estimation and identification of important mutations

In this section, genome data are preprocessed and mutations are systematically estimated to identify important mutations. For specific details of the methodology, please refer to the Methodology section.

Firstly, data are retrieved and preprocessed for downstream core estimations. Genome sequences of SARS-CoV-2 are downloaded from GISAID website. A quality examination is conducted on sequences, after which 7.7 million high-quality complete genomes are retained. Secondly, mutations on the Spike gene are identified in alignment with the reference sequence, and infrequent mutations are discarded. Then, each genome is represented by the combination of mutations, as a boolean vector representing the existence of mutations. Meanwhile, the basic reproduction number (R0) of each genome is quantified according to the variant type (Campbell et al., 2021; Liu and Rocklöv, 2021), serving as the viral fitness of SARS-CoV-2. Therefore, each genome can be represented by a boolean vector along with R0, as a row in the feature matrix. For a sound estimation, mutations observed in less than two different combinations will be discarded and finally 107 mostly frequent amino acid mutations on the Spike protein remain for further estimation. An overview of 107 studied mutations, covering their sequence statistic and R0 distribution, is illustrated in **Supplementary Figure 1**.

After the preprocessing, the core estimation on individual mutations is performed without any explicit normalization required. All the samples (rows) of the feature matrix are fed to the Linear Doubly Robust Learner for training. The average treatment effect (ATE) of each mutation is then estimated, serving as the effect score. This score represents the statistical contribution of the corresponding mutation to the viral fitness (R0) across linages. Based on the effect score of mutations, important mutations can be identified for further validations and studies. Mutations are sorted by the effect score and important mutations can be identified based on requirements such as top ten or top twenty mutations.

#### 2.1.2 Validation of mutational effects

The effect score quantifies the statistical contribution of a given mutation to the viral fitness, enabling identification of important mutations. However, the score only provides an overall estimate, and the detailed effect as well as full results from this model require validating. This section mainly validates the functional effect of identified key mutations in three major effects: Spike stability, ACE2 binding affinity, and immune escape. Computational methods are employed for these validations. Besides, relevant references are provided to supplement our understanding of mutational effects. For further information and specific details of the methodology, please refer to the Methodology section.

This section focuses on the top and bottom twenty mutations, presenting their effect scores, validated effects, and supportive references in **Table 1**. For the top twenty mutations listed in **Table 1**, except P26- mutation, possess one or more validated positive functional influences, supported by either computational validations, literature references, or both. For instance, the T478K mutation is known to stabilize the Spike protein and significantly enhance the binding affinity between Spike and ACE2 (Starr et al., 2020; Starr et al., 2022). The D614G mutation, found in VoCs since early 2020, may be involved in Spike stability, viral replication, and Spike conformation shifting, thus improving viral infectivity and transmissibility (Hou et al., 2020; Korber et al., 2020; Yurkovetskiy et al., 2020). As a key mutation in BA.2.12.1 strains (Rodino et al., 2022), S704L is another high-scoring mutation that has contributed positively across all three perspectives by computational methods of validations, indicative of its possibly compound effects, although it has not been extensively studied by scholars. Overall, most of the top twenty mutations identified in this research can possess at least one validated positive effect on viral fitness, demonstrating the effectiveness of our estimation.

**Table 1:** Effect score of the top and bottom twenty mutations, with model metrics, effect validations, and supportive references.

Mutation	VoC strains	Effect Score	P-value	MSE	Validations			
					Stability	Affinity	Escape	References
Top twenty mutations								
T478K	δ, ο	1.9298	0.0000	0.1244	+	++	−	(Starr et al., 2020; Du et al., 2021; Starr et al., 2022)
D614G	α, β, γ, δ, ο	0.6786	0.0000	0.5586	+	−	−	(Hou et al., 2020; Korber et al., 2020; Yurkovetskiy et al., 2020)
S704L	ο	0.5466	0.3050	0.7041	++	++	+	NA
H655Y	γ, ο	0.2628	0.0000	0.2689	++	++	−	(Braun et al., 2021; Zhu et al., 2022; Bloom and Neher, 2023)
N501Y	α, β, γ, ο	0.2578	0.0000	0.0495	−	+	−	(Starr et al., 2020; Du et al., 2021; Teruel et al., 2021; Starr et al., 2022; Bloom and Neher, 2023)
V213-	None	0.2332	0.1570	7.1004	NA	NA	++	NA
S477N	ο	0.1614	0.0000	0.1008	+	+	−	(Chen et al., 2020; Starr et al., 2020; Starr et al., 2022)
P26-	ο	0.1316	0.0000	0.0315	NA	NA	−	NA
Q498R	ο	0.1263	0.0000	0.0313	++	++	+	(Starr et al., 2020; Queirós-Reis et al., 2021; Starr et al., 2022)
S371F	ο	0.1120	0.0000	0.0221	+	++	+	(Miller et al., 2022; Nutalai et al., 2022)
R408S	ο	0.1114	0.0000	0.0419	−	−	−	(Sztain et al., 2021; Bloom and Neher, 2023)
T95I	ο	0.0899	0.1700	0.1056	++	−	+	(Kannan et al., 2021; Bloom and Neher, 2023)
L24-	ο	0.0795	0.0000	0.0299	NA	NA	+	NA
E484K	β, γ	0.0755	0.0050	0.0416	+	++	+	(Starr et al., 2020; Du et al., 2021; Greaney et al., 2021; Starr et al., 2022; Bloom and Neher, 2023)
T376A	ο	0.0736	0.0000	0.0356	−	−	++	NA
V213G	ο	0.0722	0.1630	0.0674	−	+	++	(Nersisyan et al., 2022)
P681H	α, ο	0.0705	0.0000	0.0725	NA	NA	++	(Haynes et al., 2021)
A222V	δ	0.0661	0.1840	0.0441	−	++	+	(Kannan et al., 2021; Bloom and Neher, 2023)
N764K	ο	0.0656	0.0000	0.0170	++	++	−	NA
D405N	ο	0.0602	0.0000	0.0301	−	+	+	(Sztain et al., 2021)
Bottom twenty mutations								
R190S	γ	-0.0087	NA	0.0266	−	−	−	NA
G446S	ο	-0.0087	NA	0.0263	−	−	+	(Cao et al., 2022; Bloom and Neher, 2023)
Q173H	None	-0.0095	NA	0.0188	−	−	+	NA
K1191N	None	-0.0120	0.4280	2.0803	NA	NA	−	NA
D80Y	None	-0.0122	0.0290	0.1972	++	−	++	NA
Y145-	None	-0.0149	0.1450	0.0263	NA	NA	−	NA
H69-	α, ο	-0.0157	0.4360	0.0444	NA	NA	−	(Kemp et al., 2021)
P681R	δ	-0.0173	0.0140	0.0841	NA	NA	++	(Liu et al., 2022)
A570D	α	-0.0187	NA	0.0426	+	+	−	NA
T572I	None	-0.0193	NA	0.0403	++	+	+	(Bloom and Neher, 2023)
R158-	None	-0.0243	NA	0.0155	NA	NA	−	NA
L18F	β, γ	-0.0265	0.0400	31.1178	−	−	++	(McCallum et al., 2021; Bloom and Neher, 2023)
S98F	None	-0.0325	NA	0.0133	−	−	++	(Bloom and Neher, 2023)
P26S	γ	-0.0337	0.2670	27.9788	−	−	++	NA
Y144V	None	-0.0361	NA	0.0255	−	+	−	NA
L5F	None	-0.1210	0.0460	0.7098	NA	NA	−	NA
W152C	None	-0.1489	0.1550	40.3863	++	−	++	(Queirós-Reis et al., 2021)
A701V	β	-0.1624	NA	1.0577	++	−	++	NA
D253G	None	-0.5367	0.4360	33.1941	NA	−	−	(Bloom and Neher, 2023)
S13I	None	-0.7669	0.1670	0.1036	NA	NA	−	(Queirós-Reis et al., 2021)

The effect score represents the statistical contribution of Spike mutations to the viral fitness. MSE represents the Mean Square Error of the corresponding model. The mutational effect is validated in the Spike protein stability, ACE2 binding affinity, the potential for immune escape, and supporting references, abbreviated as Stability, Affinity, Escape, and References, respectively. Symbol representations: ++, highly positive effect; +, potential positive effect; −, no significant positive effect; NA, not applicable. VoC strains related to each mutation are represented in Greek letters. The mutation with no validated positive effect is in red.

For a comparative study, **Table 1** also presents the bottom twenty mutations. In contrast, those mutations only possess one or no significant positive influence. Furthermore, computing analysis and literature references indicate that five mutations of the bottom twenty mutations have no significant positive effect. By comparing top and bottom twenty mutations, it becomes clear that top mutations are significantly more contributive than the bottom ones in our ranking results.

In terms of related VoCs, most of the top twenty mutations listed in **Table 1** are typical for VoCs, with the exception of the V213- mutation. Conversely, over half of the bottom twenty mutations listed in **Table 1** are not typical for VoCs. Additionally, most of the top mutations in **Table 1** have been found in Omicron strains, except for three mutations (V213-, E484K, and A222V). Accordingly, mutations of VoCs, particularly those found in Omicron variants, generally have high effect scores due to their contributions.

Based on the above discussion, we can confidently conclude that the top mutations identified by our model may be instrumental in enhancing viral fitness, potentially more so than the bottom mutations. Therefore, effect scores can effectively evaluate and identify important mutations, showing the effectiveness of this research.

#### 2.1.3 Biological study on mutant RBD

The aforementioned study utilizes causal inference models to identify the top twenty mutations with computationally validated fitness enhancements. This section further details the design of mutant RBD proteins based on those mutations and evaluates their affinity to ACE2 through biological experiments. For specific methodology details, please refer to the Methodology section.

With the chosen mutations within the RBD region, we have designed two new RBD sequences with key positions replaced by selected mutations (RBD-1: T478K, N501Y, S477N, Q498R, S371F, R408S, E484K; RBD-2: T478K, N501Y, S477N, Q498R, R408S, E484K, D405N). **Supplementary Table 2** also shows the details of mutant RBDs. Those mutant RBD proteins are intended to enhance viral fitness. In the biology laboratory, the mutant RBDs are expressed, purified, and their ACE2 binding affinity is evaluated compared to the wildtype RBD (RBD-WT).

The mutant RBD proteins are successfully expressed and purified, as demonstrated in **Supplementary Figures 2A–C**. The ACE2 affinity is estimated, with the results presented in **Supplementary Table 2** and detailed binding kinetics shown in **Supplementary Figure 2D**. In the biological experiment, the mutant RBD proteins, particularly RBD-1, exhibit stronger affinity to ACE2 than RBD-WT. Therefore, these mutation combinations are found to be contributive to the enhancement of RBD-ACE2 binding affinity and further improvement of SARS-CoV-2 viral fitness.

The computational validations, supportive references, and biological experiments described herein demonstrate the effectiveness of this study and the feasibility of further analysis. The effect score of all 107 mutations is provided as a **Supplementary file**.

### 2.2 Key mutation identification on the Spike protein

Based on the effect score of mutations, key fitness-increasing mutations can be recognized. This section aims to identify and discuss important mutations on the Spike protein, with a particular focus on the structural conformation.

Firstly, key fitness-enhancing mutations can be distinguished and studied by their quantified contributions. We utilize effect scores to identify important mutations, and further analyze them through Spike subunits and mutational occurrences, on the treemap in **Supplementary Figure 3**. **Supplementary Figure 3** illustrates mutations organized by subunits, in which the size of each rectangle represents the effect score, and the color represents the count of mutational occurrences. Generally, the overall size of the S1 subunit is considerably larger than that of S2, suggesting that the former may be more contributive to viral fitness elevation. Furthermore, it is worth noting that the effect score of mutations is not necessarily correlated with mutation count. While some long-accumulated mutations, such as D614G and T478K, play a significant role in enhancing viral fitness, others like V213- and S704L, which have emerged more recently, can still achieve high effect scores through their contributions despite fewer occurrences.

**Figure 2:**
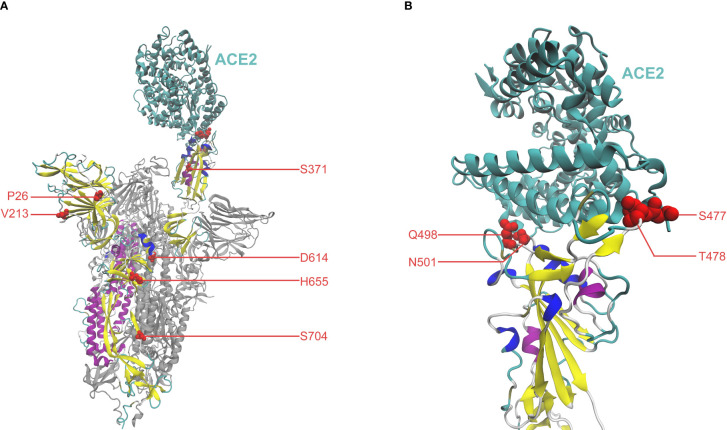
**(A)** Residues of the top ten mutations represented by red spheres, in the Spike-ACE2 complex (PDB: 7A94) (Benton et al., 2020; Wrobel et al., 2020), visualized by Visual Molecular Dynamics (VMD) (Humphrey et al., 1996; Stone et al., 2013). ACE2 is colored in cyan. The Spike monomer binding with ACE2 is colored, with the other monomers in grey. **(B)** Close-up view of the binding interface between Spike and ACE2.

Additionally, important mutations can be studied by examining their location and function on the overall structural conformation of the Spike protein. To investigate high-scoring mutations from a structural perspective, we have visualized residues of the top ten mutations in the Spike-ACE2 complex in **Figure 2**. The closed conformation of the Spike (i.e., receptor-inaccessible state) is also visualized in **Supplementary Figure 4** to facilitate a comparison study. Overall, the analysis highlights that mutations can be closely correlated with their locations and structural functions, as evidenced by literature references. Notably, four mutations (S477N, T478K, Q498R, and N501Y), occur in the binding interface between Spike and ACE2 within the receptor-binding domain (RBD, amino acid position 319-541), indicating their potential involvement in the Spike-ACE2 interaction. Supporting this notion, **Table 1** and relevant references suggest that these mutations can increase binding affinity (Chen et al., 2020; Starr et al., 2020; Queirós-Reis et al., 2021; Teruel et al., 2021; Starr et al., 2022). Another mutation of interest is S371F, which occurs in the RBD and has been reported to increase Spike stability and ACE2 affinity, and is also involved in immune escape (Queirós-Reis et al., 2021; Nutalai et al., 2022). Moreover, S371 residue may participate in the conformational transition of Spike between the open state (**Figure 2**) and closed state (**Supplementary Figure 4**), namely the up and down positions of RBD, respectively (Gur et al., 2020). Two mutations, P26- and V213-, are found within the N-terminal domain (NTD, amino acid position 14-303). NTD can be the target of human monoclonal antibodies (mAbs) (Amanat et al., 2021; McCallum et al., 2021), suggesting that these mutations could potentially contribute to the immune evasion of SARS-CoV-2 (Amanat et al., 2021; McCallum et al., 2021). For the D614G mutation, aside from its influence on Spike stability and viral replications (Hou et al., 2020; Korber et al., 2020), it can participate in the Spike conformation shift toward an ACE2 binding-competent state, before viral membrane fusion with host cells (Shang et al., 2020; Yurkovetskiy et al., 2020). In the subdomain linking S1 to S2, the H655Y mutation gives rise to a less tight loop that wraps the furin cleavage finger, thereby enhancing infectivity in the presence of N501Y (Zhu et al., 2022). In terms of S704L, despite the lack of supportive references for functional effects, validations on mutational effects by computational methods in **Table 1** have verified its effect on Spike stability, ACE2 affinity, and immune escape.

### 2.3 Regional study of the Spike protein

Different regions may have distinct functions on the Spike protein. and the mutational effect can be closely tied to these regional functions. By evaluated mutations in conjunction with their regional functions, researchers can gain a deep understanding of the subunits and domains of the Spike protein. This section conducts a regional study on the Spike protein, which researches from individual mutations to protein regions in order to recognize important mutational regions.

**Figure 3:**
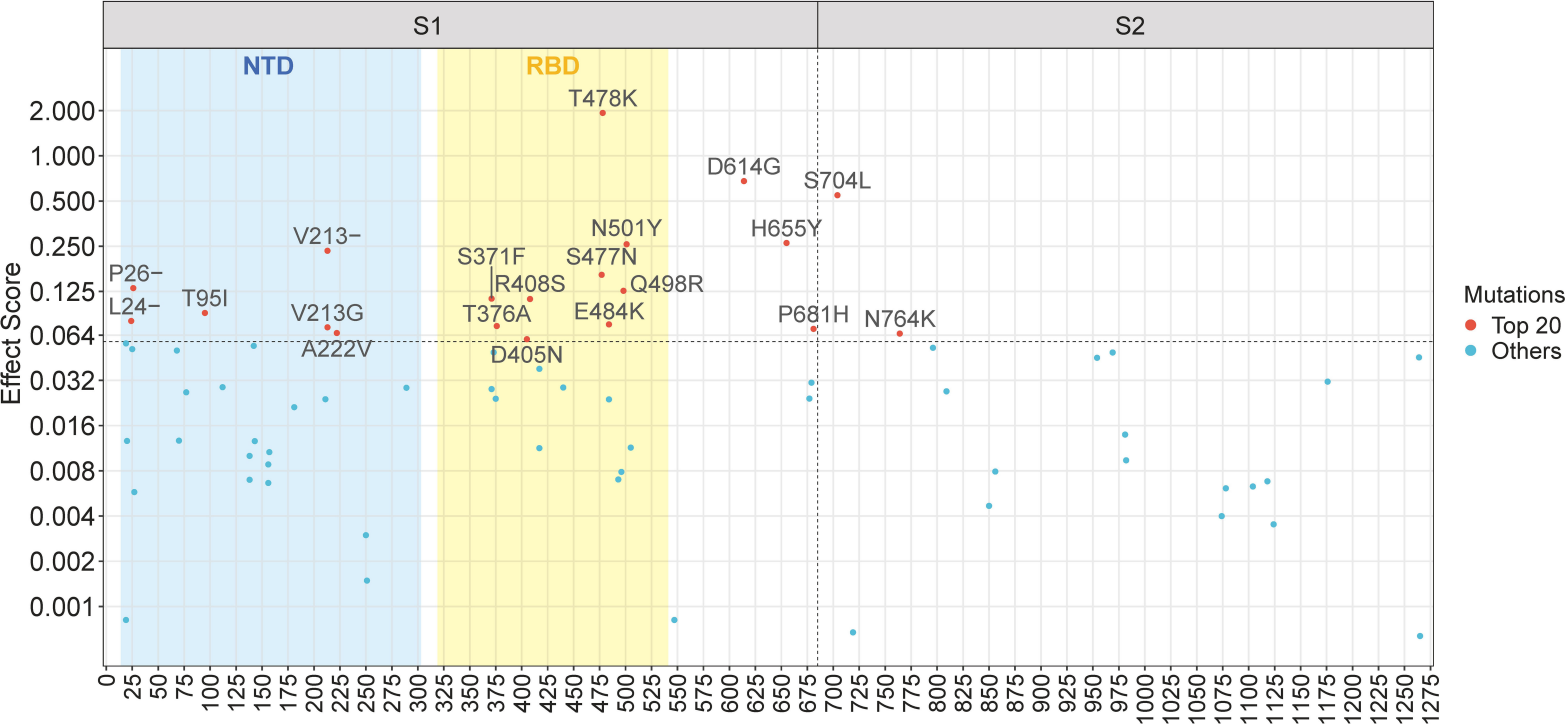
Manhattan plot of mutations with positive effect scores across the Spike gene. The top twenty mutations are explicitly labelled. The vertical dashed line represents the S1-S2 subunit boundary (amino acid position 685). The horizontal dashed line represents the lower limit of the top twenty mutations. The blue and yellow rectangles represent the region of the N-terminal domain (NTD) and the receptor-binding domain (RBD), respectively.

The Spike protein of SARS-CoV-2 consists of two subunits (see **Supplementary Figure 4**): S1 and S2, divided by the furin cleavage site at amino acid position 681-685 (Harvey et al., 2021). S1 mainly includes NTD and RBD, mediating the ACE2 binding to host cells, while S2 functionally conducts the membrane fusion with host cells (Shang et al., 2020; Harvey et al., 2021). Although S1 and S2 are both crucial to the Spike protein, they exhibit significant differences concerning mutations. For a regional study, we illustrate mutations with positive effect scores in a Manhattan plot (**Figure 3**). The plot maps mutations based on their location across the Spike gene on the x-axis and their effect scores on the y-axis. As shown in **Figure 3**, among the top twenty mutations, eighteen are clustered in the S1 subunit, indicating a greater mutational contribution by S1 compared to S2. Specifically, nine mutations occur in RBD, including the top-scoring T478K mutation. Consequently, RBD mutations are vastly important to fitness enhancement, which can be explained by its function of ACE2 binding and immune escape (Shang et al., 2020; Gaebler et al., 2021; Harvey et al., 2021). NTD likewise plays a part in viral infection and contains six high-scoring mutations. Notably, some important mutations, such as H655Y and P681H, are located near the S1-S2 subunit boundary, which may be related to the furin cleavage site (Zhu et al., 2022) and facilitate the conformational shift of Spike (Harvey et al., 2021). In contrast to S1, mutations in S2 generally have modest effect scores, with the exception of S704L and N764K.

To explore the distribution of effect scores in different regions, we have also displayed the effect score of mutations organized by subunits/domains, in **Supplementary Figure 5**, including both positive and negative scores. Despite the approximate length of S1 and S2, S1 has considerably more mutations, especially high-scoring ones, making it more contributive to fitness elevation. Conversely, the S2 subunit can be considerably more conserved with fewer mutations compared with S1 (Shah et al., 2021). Of the 81 mutations in S1, 46 mutations are concentrated in NTD, but most scores for mutation in NTD are modest. Compared with other regions, RBD generally has a higher distribution of effect scores. Due to its crucial function, RBD serves as an important domain in the fitness enhancement of SARS-CoV-2.

### 2.4 Viral fitness and R0 prediction

Evaluated individual mutations can provide an estimate of the fitness score for different SARS-CoV-2 strains. This section makes further efforts to explore the viral fitness and R0 prediction of SARS-CoV-2 strains using effect scores.

Since individual mutation has been evaluated, the fitness score for a given sequence can be defined as the sum of effect scores for its mutations. The original Wuhan strain (wildtype) has a fitness score of zero, which serves as a baseline for the fitness score.

**Figure 4:**
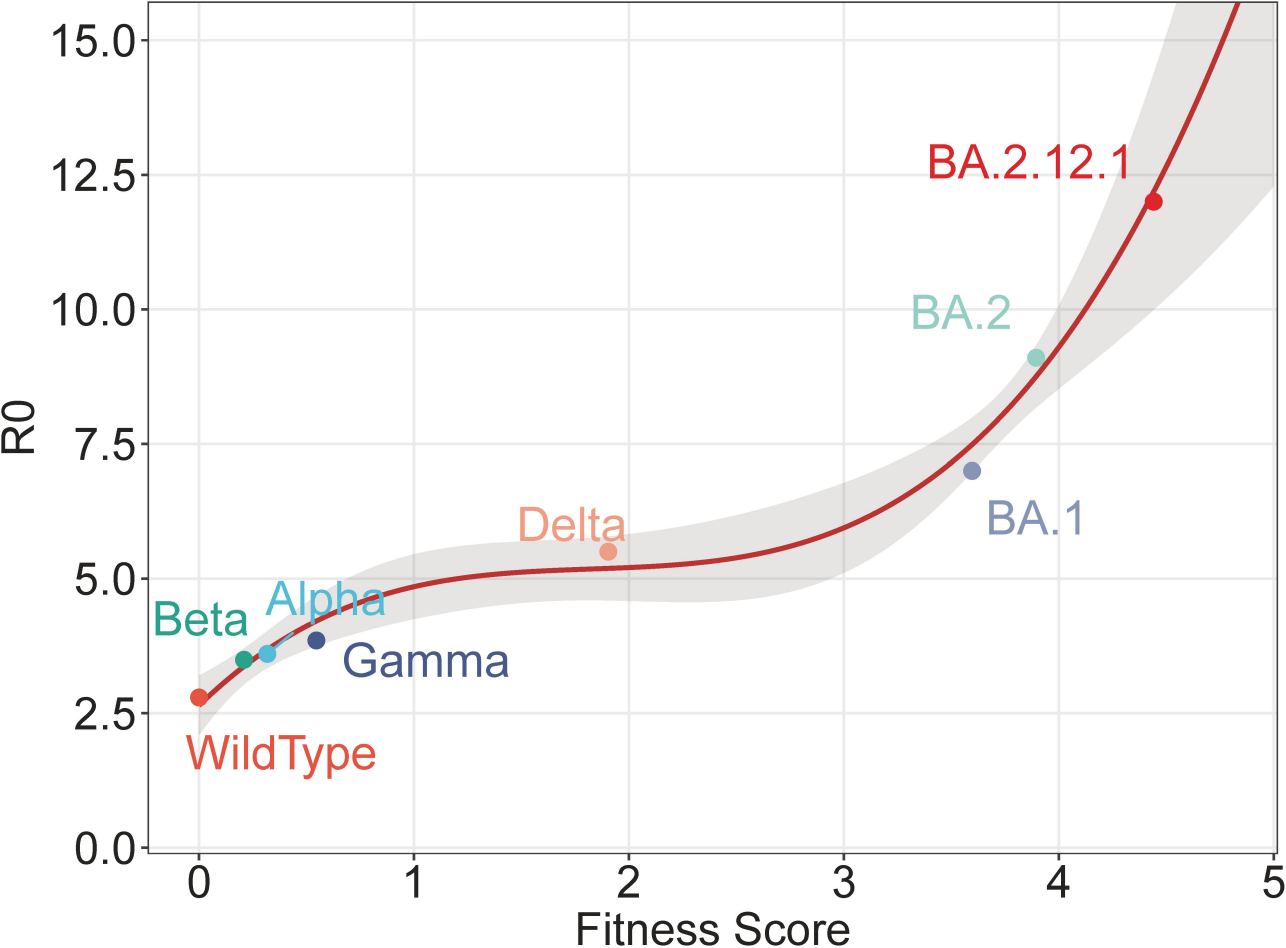
Polynomial regression of fitness score and R0. The regression is trained by all points except BA.2.12.1, with the shaded area representing the 75% confidence interval. The point of BA.2.12.1 is subsequently illustrated as a validation.

We compare the fitness score and R0 for both the wildtype and VoCs in **Supplementary Figure 6**. **Supplementary Figure 6** demonstrates that the rank by the fitness score is consistent with that of R0, indicating a correlation between the two sides. To visualize the correlation, we have further plotted those strains as points in **Figure 4**, in which the x-axis represents the fitness score and the y-axis shows the R0 value. The plot reveals a clear correlation, which can be represented by a regression line. All points except subsequent BA.2.12.1 are used to train a polynomial regression (with degree 3). These points are generally located within the 75% confidential interval (CI) and are close to the regression line. The regression line after BA.2 predicts values other than training. As a validation, the value of BA.2.12.1 is subsequently plotted, which fits well with the predicted values. This close agreement between the predicted line and the validation BA.2.12.1 demonstrates the effectiveness of the fitness score in predicting R0.

**Figure 5:**
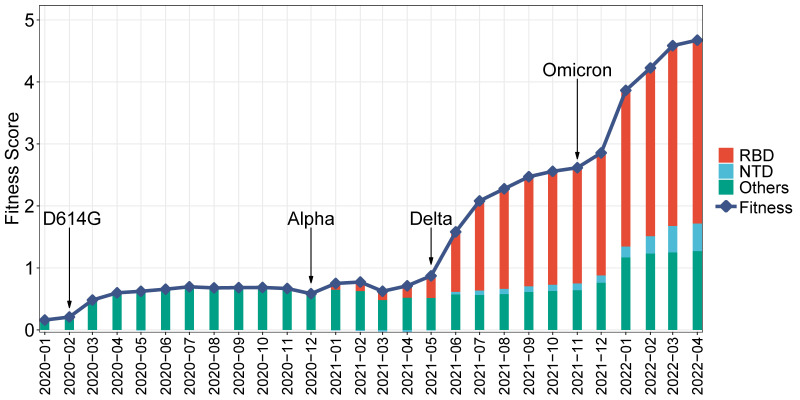
Monthly historical fitness score of SARS-CoV-2. The line represents the historical fitness score. Stacked bars show the contribution of different Spike regions. The emergence of strains is indicated: the D614G strain, Alpha, Delta, and Omicron.

Further, the historical fitness score of SARS-CoV-2 is explored. Given the many strains that have emerged during each period, the historical fitness score is calculated as the overall fitness during a specific period. This score is determined by the weighted sum of effect scores, with the weight being the mutation frequency during that time period. The historical fitness score from January 2020 to April 2022 is presented in **Figure 5**, along with the contribution of RBD and NTD. This figure demonstrates a steady increase in the historical viral fitness score during the pandemic, which coincides well with the contemporaneous emergence of VoCs. For instance, the D614G strain rose to prominence in Feb 2020 (Korber et al., 2020), leading to an increase in fitness at that time. Similar increases can be observed with the emergence of the Alpha, Delta, and Omicron variants, respectively. The viral fitness increase has been accelerating over time, especially since the emergence of Delta and Omicron. As for Spike regions, the contribution of RBD has significantly increased, from being a minority in 2020 to becoming the majority since mid-2021. Similarly, the contributions of NTD and other regions have increased, although not to the same extent as RBD.

## 3 Discussion

The Spike protein is of paramount importance to the viral fitness of SARS-CoV-2, especially in terms of transmissibility (Shang et al., 2020; Gaebler et al., 2021; Harvey et al., 2021), and has been extensively studied. This manuscript concentrates on estimating the statistical contribution of Spike mutations and identifying key Spike mutations for viral fitness. While individual mutations such as D614G (Hou et al., 2020; Korber et al., 2020; Yurkovetskiy et al., 2020) and N501Y (Starr et al., 2020; Teruel et al., 2021; Starr et al., 2022) have been studied, extensive assessment of mutational contributions in the context of large-scale genomes still remains challenging. The challenge lies in the fact that SARS-CoV-2 mutations often act as confounding factors to each other, making evaluations on an individual mutation a challenging issue. However, causal inference, one of the most promising methods in data science, produces an unbiased estimation of the treatment effect on outcomes, as a function of observable characteristics of samples (Pearl, 2009; Pearl and Mackenzie, 2018; Guo et al., 2020; Yao et al., 2021). It is designed to solve the statistical problem in which variables are confounding factors to each other, and is therefore naturally applicable to the mutational analysis of SARS-CoV-2. With the help of causal inference models, Spike mutations can be evaluated, identified, and utilized for further analysis.

To employ causal inference models, a quantitative phenotype is required for representing viral fitness as the outcome variable. In this paper, R0 is chosen as the outcome variable because it clearly reveals viral transmissibility, which is one of the most important quantitative phenotypes for viral fitness. Moreover, the estimation of R0 for SARS-CoV-2 variants has been extensively studied and widely recognized (Campbell et al., 2021; Liu and Rocklöv, 2021). Therefore, R0 can be considered a representative and qualified measure of viral fitness. Likewise, other phenotypes of SARS-CoV-2 can be used for causal inference models as long as quantitated. Hence, this work can be transferred and applied to other quantitative phenotypes, and even to other viral genomic data.

It is worth noting that the estimation of causal inference models only provides an effect score for each mutation, and the detailed mutational effect is supposed to be validated and interpreted by other methods. To confirm the functional effect of significant mutations, this study utilizes computational methods to explore mutational effects in three major aspects: Spike stability, ACE2 affinity, and immune escape. As demonstrated in the Results section, the top twenty mutations exhibit more contributive effects than the lower-ranked ones, and thus they can be more important to viral fitness. Moreover, the validation results are consistent with existing literature references. This consistency provides a solid cornerstone for the validation and explanation of detailed effects of identified critical mutations, and further demonstrates the effectiveness of effect scores.

Although this paper includes three important mutational effects in validations, there are still other aspects of effects, such as viral replication and viral pathogenicity. Therefore, it is important to note that a mutation with no validated influence in **Table 1** does not necessarily imply no positive effect at all. To provide supplementary validation, literature references have also been included. For instance, while the R408S mutation shows no remarkable influence by computing validations in **Table 1**, previous research suggests that it may facilitate the opening of RBD (Sztain et al., 2021). Another noticeable mutation is S704L, which is positively influential in **Table 1** by computing results. Further investigation is needed as S704L has not been thoroughly investigated, making it an interesting direction for future research.

It is worth noting that the primary focus of mutational effect research may have shifted. For early mutations like D614G, existing literatures has concentrated on how it increases viral transmissibility (Hou et al., 2020; Korber et al., 2020), by affecting Spike stability and viral replications, etc. In contrast, for newly-emerged mutations, especially those exclusive to Omicron strains, such as G446S and R493Q, relevant studies primarily focus on their mutational effects on the immune escape, particularly antibody evasion (Cao et al., 2022; Iketani et al., 2022; Wang et al., 2022). This shift in focus can be attributed to the increased significance of immune evasion. On the one hand, a growing number of individuals have possessed antibodies against SARS-CoV-2 over time, either through infection or vaccination, which may potentially increase the selection pressure for SARS-CoV-2. On the other hand, the emergence and spread of diverse variants of SARS-CoV-2 highlight the importance of studying the efficacy of current vaccines in protecting against the virus.

Another interesting phenomenon is that the effect of Spike mutations can be highly correlated with the protein region. For instance, RBD functionally conducts ACE2 binding (Shang et al., 2020) and can be the target of antibodies (Gaebler et al., 2021). Consequently, many mutations that enhance ACE2 binding affinity and enable immune evasion occur in the RBD region, making it a region with high-scoring mutations. Recently emerged RBD mutations, such as R346K, F486V, and R493Q have been found to be closely related to antibody evasion in Omicron subvariants like BA.2.12.1 and BA.4/5, making RBD one of the most important regions for mutational effects (Iketani et al., 2022; Wang et al., 2022). Another domain, NTD, has the highest number of mutations, but their average scores are modest, so they may be not vastly noticeable. Although some mutations in NTD, e.g., T95I, V213G, and A222V, may be involved with immune escape (Kannan et al., 2021; Nersisyan et al., 2022), the specific function of NTD and its mutations still remain to be elucidated. Besides, some important mutations locate at or near the furin cleavage site. As the furin cleavage site is essential to SARS-CoV-2 (Harvey et al., 2021; Johnson et al., 2021), these mutations may have a functional correlation with it, like P681R (Liu et al., 2022) and H655Y (Zhu et al., 2022).

Interestingly, the S1 and S2 subunits can be entirely different when it comes to mutations. As depicted in **Figure 3**, S1 appears to be more prone to high-scoring mutations, while S2 tends to be relatively conserved with fewer mutations. While both subunits possess important functions for the Spike protein, such as receptor-binding for S1 and membrane fusion for S2, it is difficult to determine which side holds greater importance. Nevertheless, these two subunits demonstrate entirely different mutation tendencies. This phenomenon may be an interesting issue for further studies. One possible explanation for this pattern could be due to the fact that the S1 subunit is usually the target of antibodies (Amanat et al., 2021; Gaebler et al., 2021; McCallum et al., 2021), thus allowing for multiple mutations to occur for viral immune evasion. On the other hand, the conservation of S2 may also make it a potential target of medicine and general vaccine development against the rapid immune escape of SARS-CoV-2 (Shah et al., 2021).

In the present study, the contributions of mutations are learned from R0, and conversely, reveal the relative viral fitness. However, during the model training, BA.2.12.1 strains are not specifically distinguished and are recognized as ordinary BA.2 strains. Despite this, BA.2.12.1 still achieves a higher fitness score than BA.2, hence the fitness score can effectively reveal viral fitness. Additionally, the regression in **Figure 4** is not trained using BA.2.12.1 strains, but BA.2.12.1 fits well within the regression, which validates the effectiveness of the model. Therefore, for an unknown strain of SARS-CoV-2, its relative fitness and R0 can be computationally predicted, solely based on its viral sequence, by computing its fitness score by mutations and further estimating R0 according to the regression. This capability of prediction is significant for monitoring and prewarning newly-emerged strains of SARS-CoV-2.

One interesting phenomenon in **Figure 5** is the synchronization between the increase in historical fitness and the emergence of variants. The driving forces behind may be related to the selective sweep of SARS-CoV-2 (Wang et al., 2022), in which previous predominant strains are swept and replaced by new ones. During the selective sweep, the prevalence of new variants implies possible adaptive advantages compared with previous ones, which can lead to a higher fitness score. According to the upward trend of the regression line in **Figure 4**, future strains with higher scores may enjoy significantly enhanced vial fitness such as transmissibility and immune escape, which may further prolong the pandemic. Accordingly, it is important to strengthen epidemiological surveillance of new SARS-CoV-2 variants.

Causal inference models can also provide secondary information through interpretability. By using other covariates mutations as features, SHAP values can interpret the model of each mutation and the relation between the current mutation and others (Lundberg and Lee, 2017). **Supplementary Figure 7** interprets the model of the top mutations, which represent the unidirectional influence of other mutations on the object mutation. For mutations that are mutually top influential to each other, their bidirectional influences may reveal possible mutation interactions. These interactions are categorized into positive and negative ones, according to the mutational co-occurrence and exclusion, respectively. **Supplementary Figure 8** illustrates the possible interactions discovered in this study, which require further investigation and confirmation.

## 4 Methodology

This work is schematically depicted in **Figure 1**, consisting of the Data Preprocessing, the Effect Estimation, the Validation and Application, etc.

### 4.1 Data preprocessing

#### 4.1.1 Data source and data curation

From GISAID Website (Shu and McCauley, 2017), SARS-CoV-2 complete genome sequences as of 11 May 2022 are retrieved. These genome sequences are presented as FASTA files of bases (A, C, G, and T), and undergo quality examination. The examination involves two criteria:

1. Completion - the sequence must be longer than 80% of the length of the reference sequence.
2. High-quality - the percentage of invalid characters should be below 10%.

Only retrieved sequences that meet both criteria are retained, while others are discarded. As such, 7,699,174 high-quality complete genome sequences remain for downstream analysis (refer to Supplementary_FastaID.csv for additional information). For each genome, amino acid mutations on the Spike gene are identified in alignment with the reference sequence Wuhan-Hu-1 (GenBank accession number NC_045512). Spike mutations with global occurrence of less than 1,500 are considered infrequent and are consequently discarded.

#### 4.1.2 Feature extraction

The feature matrix is generated to represent Spike sequences by mutation combinations and R0. Each Spike sequence is mapped into a mutation combination, and then represented by a boolean vector of Spike mutations, as a row vector in the feature matrix, along with R0 according to the variant type (Campbell et al., 2021; Liu and Rocklöv, 2021). **Supplementary Table 3** provides detailed information on how R0 is assigned based on previous studies (Campbell et al., 2021; Liu and Rocklöv, 2021). Mutation combinations with either a global occurrence of less than 1500 or an inestimable R0 are excluded from the analysis. Since replicate rows in the feature matrix do not affect the unbiased estimation of causal inference, redundant rows are merged. To ensure accurate estimation, mutations are supposed to be observed in at least two mutation combinations, otherwise they will be discarded. Consequently, 107 major amino acid Spike mutations are retained for further studies. Note that while this study focuses on these 107 mutations, additional mutations may be considered as long as they pass the preprocessing examination.

### 4.2 Effect estimation

Mutations are modelled and evaluated successively. Specifically, Linear Doubly Robust Learner (Linear DRL) (Bang and Robins, 2005; Dudík et al., 2014) is employed, with an assumption of linear form of treatment effect (Bang and Robins, 2005; Dudík et al., 2014). For each mutation, a Linear DRL is utilized for an estimation of its effect across lineages. For a given mutation *M_i_* as treatment *T*, its effect score is estimated by *θ_T_*, namely the average treatment effect (ATE) on outcomes, with R0 as the observed outcome *Y* and other mutations as observable characteristics (covariates) *X* on samples. The estimation is based on all the observed i.i.d. samples from the feature matrix, with the *j*-th row being the sample (*Y_j_*,*T_j_*,*X_j_*). This approach uses all available samples for the model training, without requiring explicit normalization or a separate validation or testing set.


$$\theta_{T}=E\left[Y^{\left(T=1\right)}-Y^{\left(T=0\right)}|X\right]$$

### 4.3 Validation of mutational effects

This study employs computing methods to validate the functional influences of mutations, including Spike protein stability, ACE2 binding affinity, and potential for immune escape.

#### 4.3.1 Spike protein stability

FoldX5 (Schymkowitz et al., 2005) is performed to estimate mutational effects on the stability of Spike protein in closed conformation (PDB: 7DDD) (Zhang et al., 2021). Specifically, FoldX5 evaluates quantitative changes in the Gibbs energy of protein folding caused by mutations (ΔΔG, unit: kcal/mol) (Schymkowitz et al., 2005). Mutation effects on ΔΔG include highly positive effect (ΔΔG< −1.0), potential positive effect (−1.0< ΔΔG< 0), and no significant positive effect (ΔΔG > 0). Indels (insertions and deletions) and mutations at unmodeled residues of the protein are inapplicable to FoldX5 and are labelled as NA (not applicable).

#### 4.3.2 ACE2 binding affinity

FoldX5 (Schymkowitz et al., 2005) is performed to estimate mutational effects on the Spike-ACE2 complex (PDB: 7A94) (Benton et al., 2020; Wrobel et al., 2020). FoldX5 evaluates quantitative changes in the Gibbs energy caused by mutations (ΔΔG, unit: kcal/mol) (Schymkowitz et al., 2005). Mutation effects on ΔΔG include highly positive effect (ΔΔG< −1.0), potential positive effect (−1.0< ΔΔG< 0), and no significant positive effect (ΔΔG > 0). Indels (insertions and deletions) and mutations at unmodeled residues of the protein are inapplicable to FoldX5 and are labelled as NA (not applicable).

#### 4.3.3 Immune escape

A system named Constrained Semantic Change Search (CSCS) (Hie et al., 2021) is utilized to estimate semantic changes (Δs) of SARS-CoV-2 Spike sequence for the potential for immune escape caused by mutations (Hie et al., 2021). Mutation effects on semantic changes include highly positive effect (Δs > 0.9), potential positive effect (0.75< Δs< 0.9), and no significant positive effect (Δs< 0.75).

### 4.4 Biological study on mutant RBD

For biological experimental investigations, mutant RBD proteins based on top RBD mutations in **Table 1** are expressed, purified, and then evaluated for ACE2 binding affinity, compared with the wildtype RBD.

#### 4.4.1 RBD expression and purification

The wildtype and mutant RBD proteins are expressed and purified by the method in prior works (Liu et al., 2022). Mutant RBDs are designed by mutation sites into pPICZαA-RBD-WT, according to the mutation in **Supplementary Table 1**. The plasmids of pPICZαA-RBD are linearized by BglII and transformed into the glycoengineered yeast (Liu et al., 2020). Positive clones of RBD are screened by western blot analysis. After the shake-flask culture, the product is centrifuged at 8500× g rpm for 15 min. The harvested supernatant is purified as described previously (Liu et al., 2022). Purified proteins are analyzed by sodium dodecyl sulfate–polyacrylamide gel electrophoresis (SDS-PAGE).

#### 4.4.2 RBD-ACE2 affinity

The binding kinetics of RBDs to His-tagged human angiotensin‐converting enzyme 2 (ACE2) is assayed and evaluated by the ForteBio Octet™ QKe System (Pall ForteBio Corporation) (Guo et al., 2022). RBDs and ACE2 are diluted to 400 nM with HBS-EP (Cytiva), and an additional well with only HBS-EP is set up as a control. ACE2 is bound to the probe capturing the His tag. After the stabilization of RBD-ACE2 binding, the dissociation is performed in HBS-EP. The dissociation constant (Kd) is calculated by Data Analysis Software 7.0 (Pall ForteBio Corporation).

## 5 Conclusions

This manuscript proposes and formulates a well-defined framework of an unbiased approach for evaluating and identifying key Spike mutations of SARS-CoV-2 by causal inference models, in the context of large-scale genomes. By analyzing 7.7 million viral genome sequences, this study evaluates the contribution of mutations to viral fitness across lineages, and identifies important mutations accordingly. As validated, high-scoring mutations possess one or more positive mutational effects, which demonstrates the effectiveness of this research. Based on the effect score, key fitness-enhancing mutations and protein regions have been studied. Notably, RBD mutations play an important role in the fitness elevation of SARS-CoV-2. Besides, the fitness and R0 of unknown SARS-CoV-2 strains can be predicted, solely based on the viral sequence. This approach provides reliable guidance about mutations of interest, including some high-scoring but less-studied mutations like S704L. Moreover, the present work can be transferred to other quantitative phenotypes of SARS-CoV-2 for evaluating specific mutational effects, e.g., immune escape. Overall, this approach produces innovative and systematic insights into SARS-CoV-2 mutations, which may contribute to the evolutionary characterization of SARS-CoV-2 and the development of Spike-targeted medicines and vaccines against SARS-CoV-2. As the first application of causal inference models to mutational analysis on SARS-CoV-2 genomes, this work may inspire more related applications and promote the development of interdisciplinary fields.

## Data availability statement

The datasets presented in this study can be found in online repositories. The information and GISAID accession number(s) of genome sequences can be found in Supplementary_FastaID.csv at https://github.com/Dywangxin/spikemut.

## Author contributions

HR, JY, and JW formulated the study. XW and MH performed the research and analyzed the data. YJ, BW, and YZ participated in analysis and discussion. XW and HR drafted the manuscript. BL and HX conducted the biological investigation. All authors contributed to the article and approved the submitted version.
