# Supplementary material for: Evaluating the effect of SARS-CoV-2 spike mutations with a linear doubly robust learner
Source: Front Cell Infect Microbiol. 2023 Apr 19;13:1161445. doi: 10.3389/fcimb.2023.1161445 (PMC10154619; doi:10.3389/fcimb.2023.1161445)
Supplement: Supplementary file 1 [file DataSheet_1.docx]

Supplementary Material

Evaluating the effect of SARS-CoV-2 spike mutations with a linear doubly robust learner

Xin Wang^†^, Mingda Hu^†^, Bo Liu^†^, Huifang Xu, Yuan Jin, Boqian Wang, Yunxiang Zhao, Jun Wu^*^, Junjie Yue^*^, Hongguang Ren^*^

**† Contributed Equally.**

*** Correspondence:**  Hongguang Ren, bioren@163.com; Junjie Yue, yue_junjie@126.com; Jun Wu, junwu1969@163.com

# Supplementary Tables

**Supplementary Table 1.** List of the investigated proteins of the receptor-binding domain (RBD). RBD-1 and RBD-2 are both mutant proteins; RBD-WT represents the wildtype.

| **RBDs** | **Mutations** |
| --- | --- |
| RBD-1 | T478K, N501Y, S477N, Q498R, S371F, R408S, E484K |
| RBD-2 | T478K, N501Y, S477N, Q498R, R408S, E484K, D405N |
| RBD-WT | None |

**Supplementary Table 2.** The human angiotensin-converting enzyme 2 (ACE2) binding affinity of studied proteins of the receptor-binding domain (RBD): K_D_, equilibrium disassociation constant; K_a_, association rate constant; K_d_, disassociation rate constant. (n=3)

| **RBDs** | **Mean** | | | |
| --- | --- | --- | --- | --- |
|  | **K_D_(M)** | **K_a_(10^5^Ms^-1^)** | **K_d_(s^-1^)** | **x^2^(RU^2^)** |
| RBD-1 | 4.15×10^-10^ | 3.47 | 1.44×10^-4^ | 0.005 |
| RBD-2 | 4.06×10^-9^ | 4.12 | 1.67×10^-3^ | 0.009 |
| RBD-WT | 3.14×10^-8^ | 2.90 | 9.10×10^-3^ | 0.006 |

**Supplementary Table 3.** Basic reproduction number (R0) of SARS-CoV-2 strains used for the effect score estimation. Abbreviations: VoC, Variant of Concern; R0, basic reproduction number.

| **Variant** | **Is VoC** | **R0** |
| --- | --- | --- |
| WildType | No | 2.79 |
| Alpha | Yes | 3.60 |
| Beta | Yes | 3.49 |
| Gamma | Yes | 3.85 |
| Iota | No | 3.80 |
| Epsilon | No | 3.01 |
| Kappa | No | 4.13 |
| Delta | Yes | 5.50 |
| Omicron BA.1 | Yes | 7.00 |
| Omicron BA.2 | Yes | 9.10 |

# Supplementary Figures


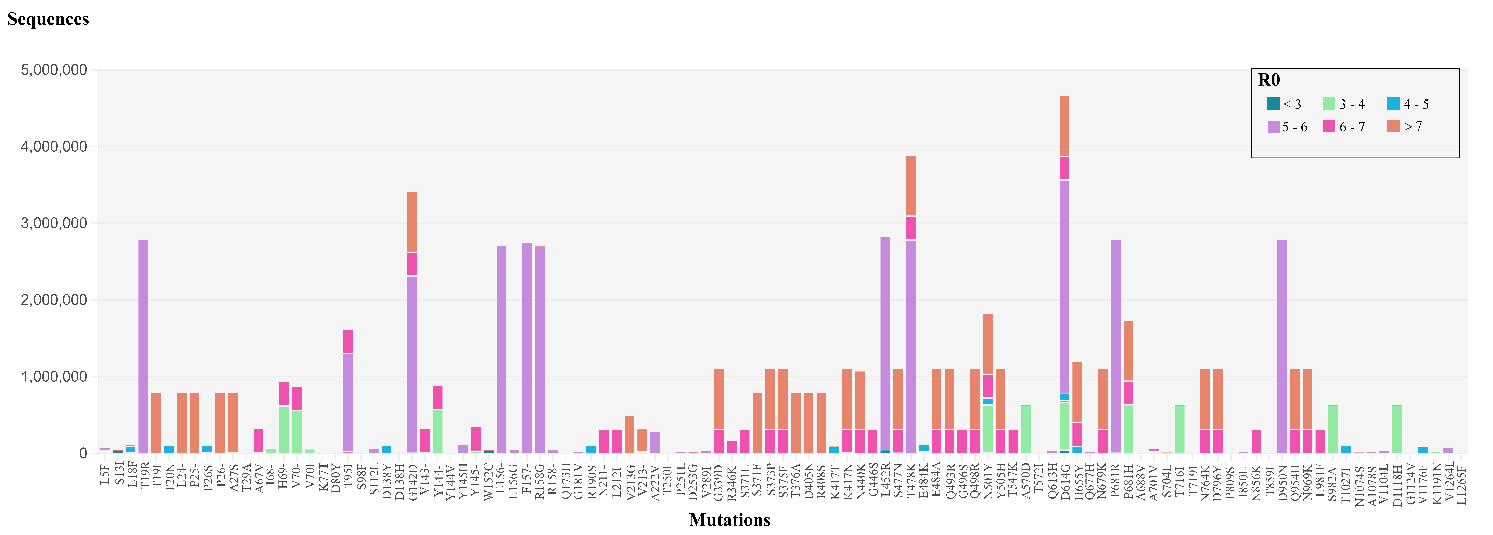


**Supplementary Figure 1.** Sequence statistic and R0 (basic reproduction number) distribution of 107 studied mutations. The color denotes the R0 value of corresponding strains. The height of bars represents the count of mutational occurrences. Mutations are illustrated respectively.


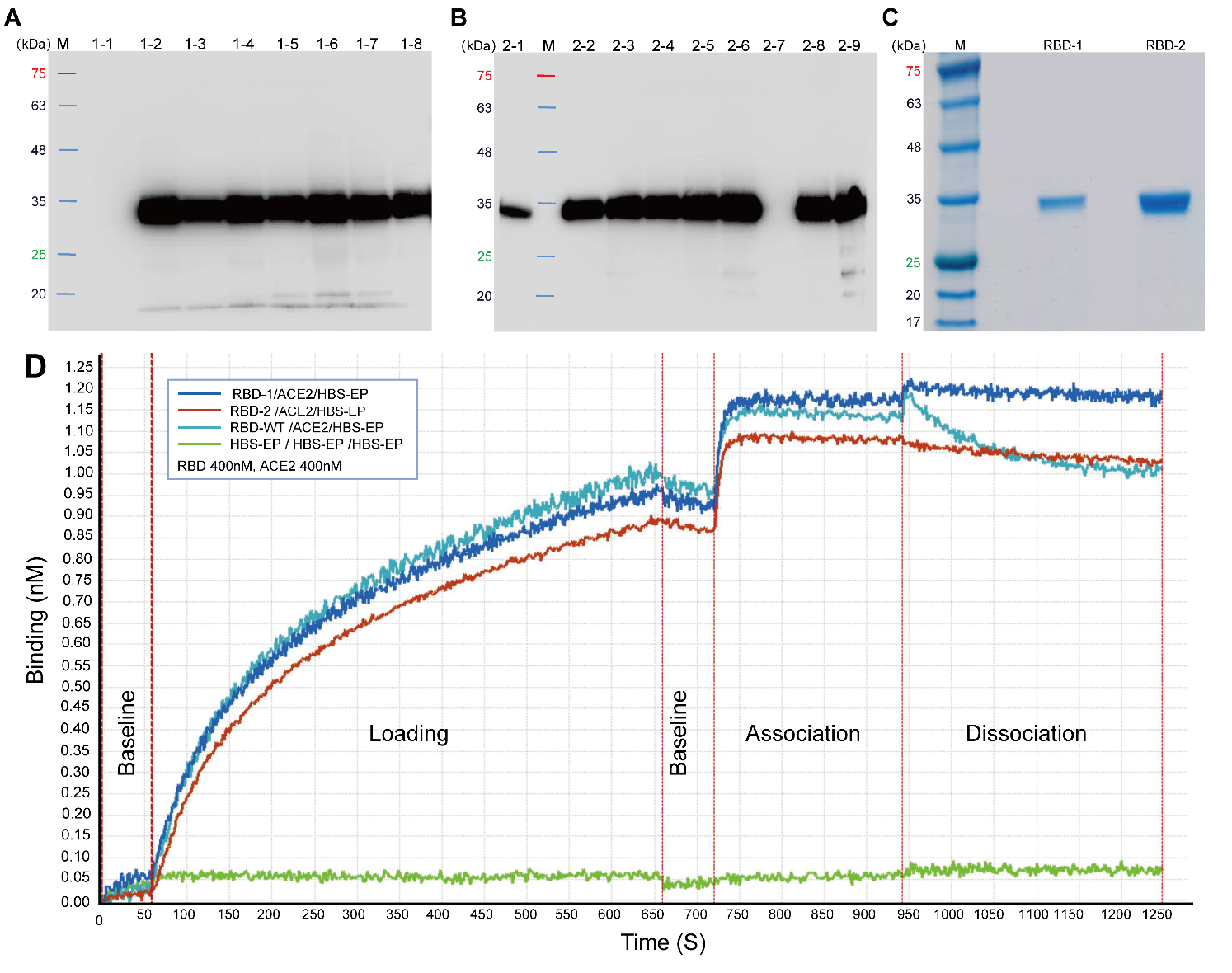


**Supplementary Figure 2.** Expression, purification, and binding kinetics of mutant RBD proteins. **(A)** Western blot analysis of various glycoengineered clones of RBD-1. **(B)** Western blot analysis of various glycoengineered clones of RBD-1. **(C)** Sodium dodecyl sulfate–polyacrylamide gel electrophoresis (SDS-PAGE) analyses of RBD-1 and RBD-2 proteins. **(D)** RBD-ACE2 binding kinetics of studied proteins of the receptor-binding domain (RBD). In the legend box, the samples separated by slash “/” represent stages of sample molecules: Loading, Association, and Dissociation, respectively.


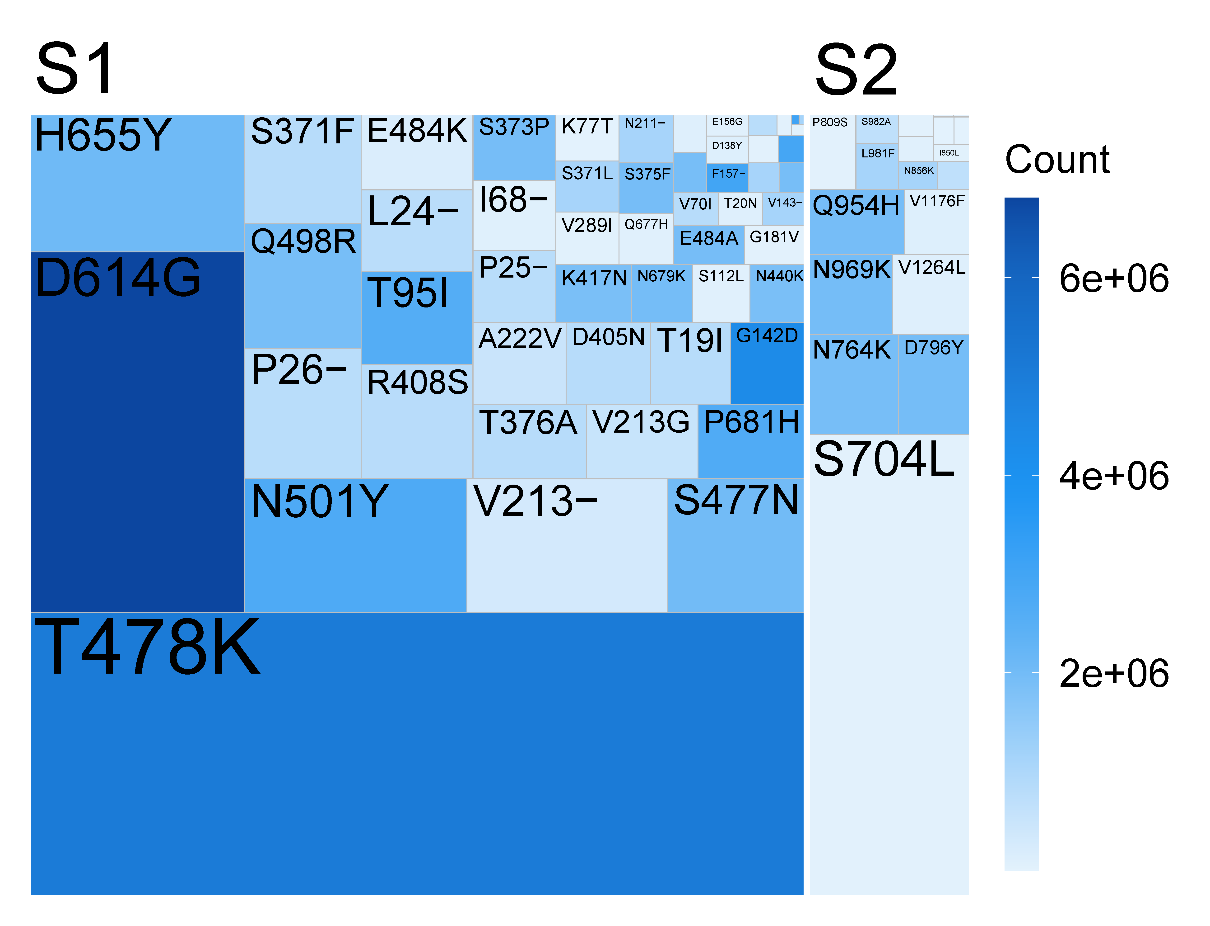


**Supplementary Figure 3.** Treemap of Spike mutations. The size represents the effect score of mutations, and the color represents the count of mutational occurrences. Mutations of the S1 and S2 subunits are grouped respectively.


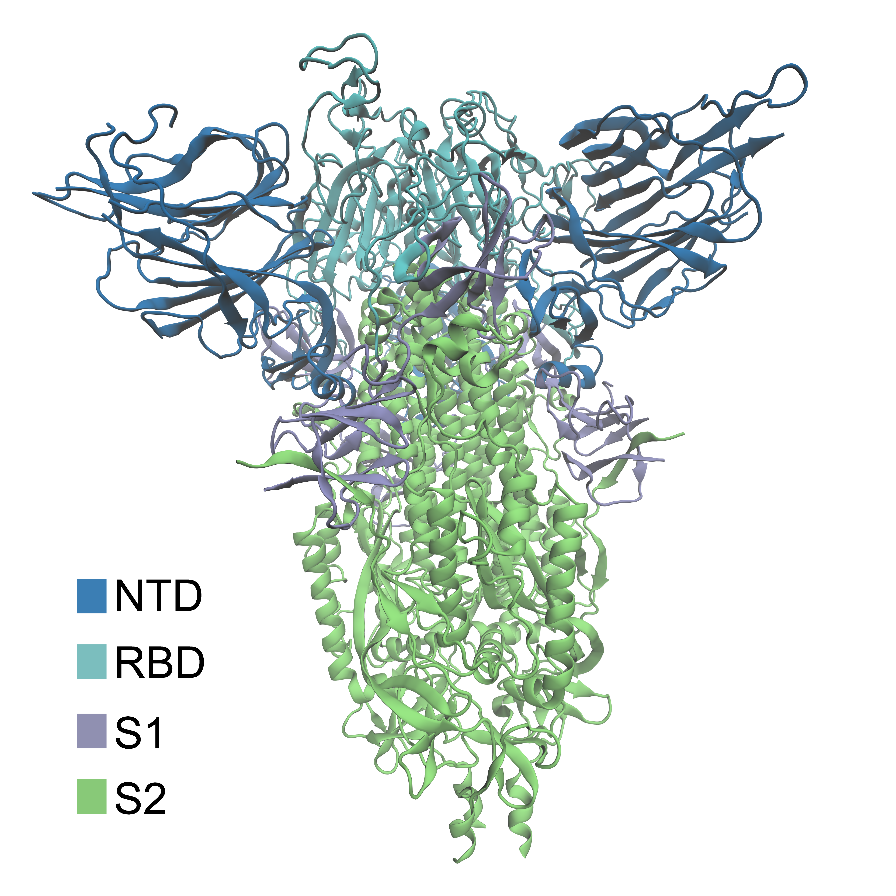


**Supplementary Figure 4.** Structure of the Spike protein in closed conformation (PDB: 7DDD) [7], visualized by Visual Molecular Dynamics (VMD) [8, 9]. Colors represent different domains and subunits: blue, the N-terminal domain (NTD); cyan, the receptor-binding domain (RBD); grey, the S1 subunit; green, the S2 subunit. Note that RBD and NTD are particularly distinguished from other parts of the S1 subunit by different colors.


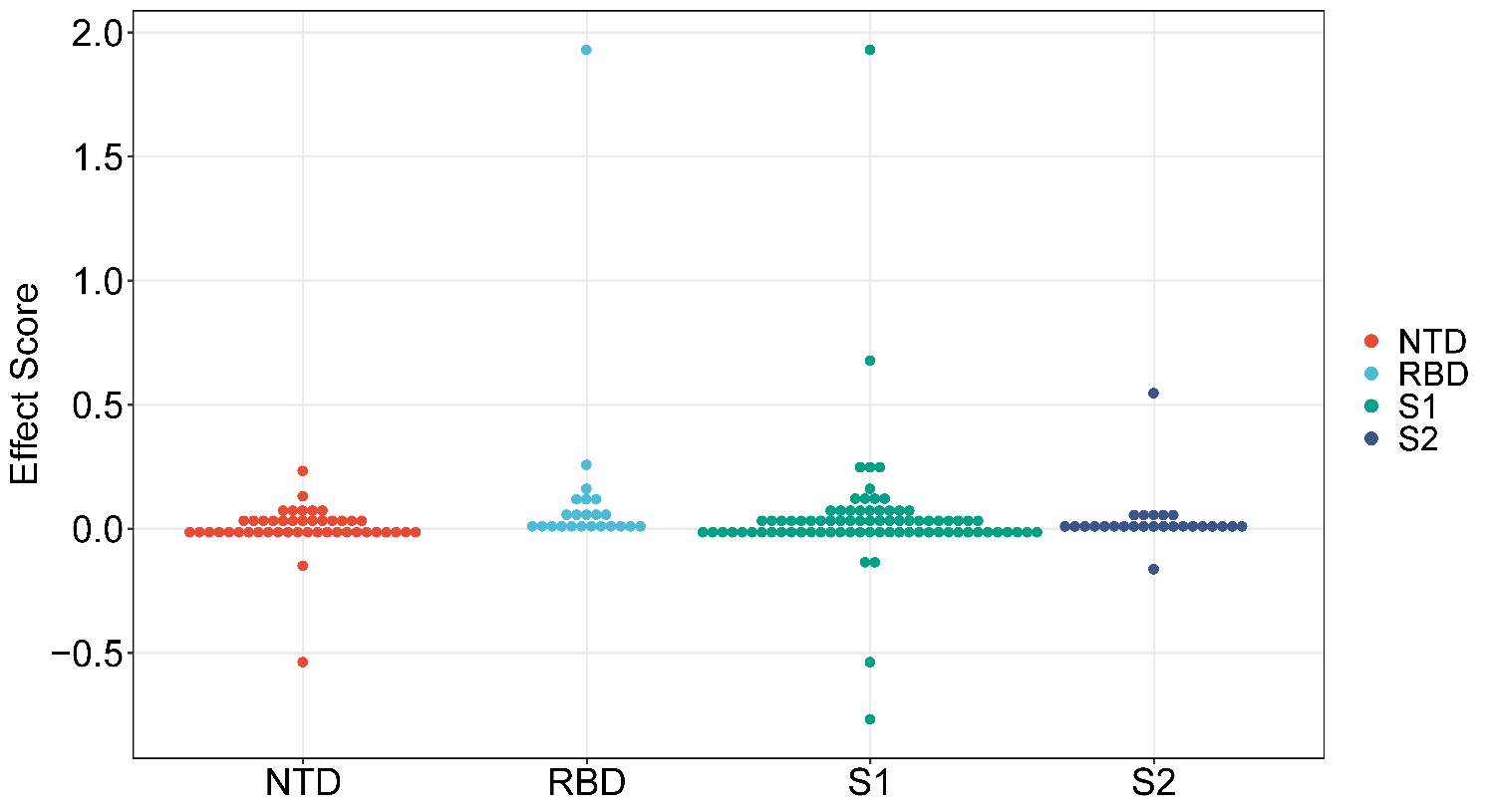


**Supplementary Figure 5.** Dot plot of effect scores of mutations, grouped by different subunits and domains: the N-terminal domain (NTD), the receptor-binding domain (RBD), the S1 subunit, and the S2 subunit. Dot-density binning is applied, with each dot representing one or two close observations. Note that mutations of RBD and NTD are also included in the S1 subunit.


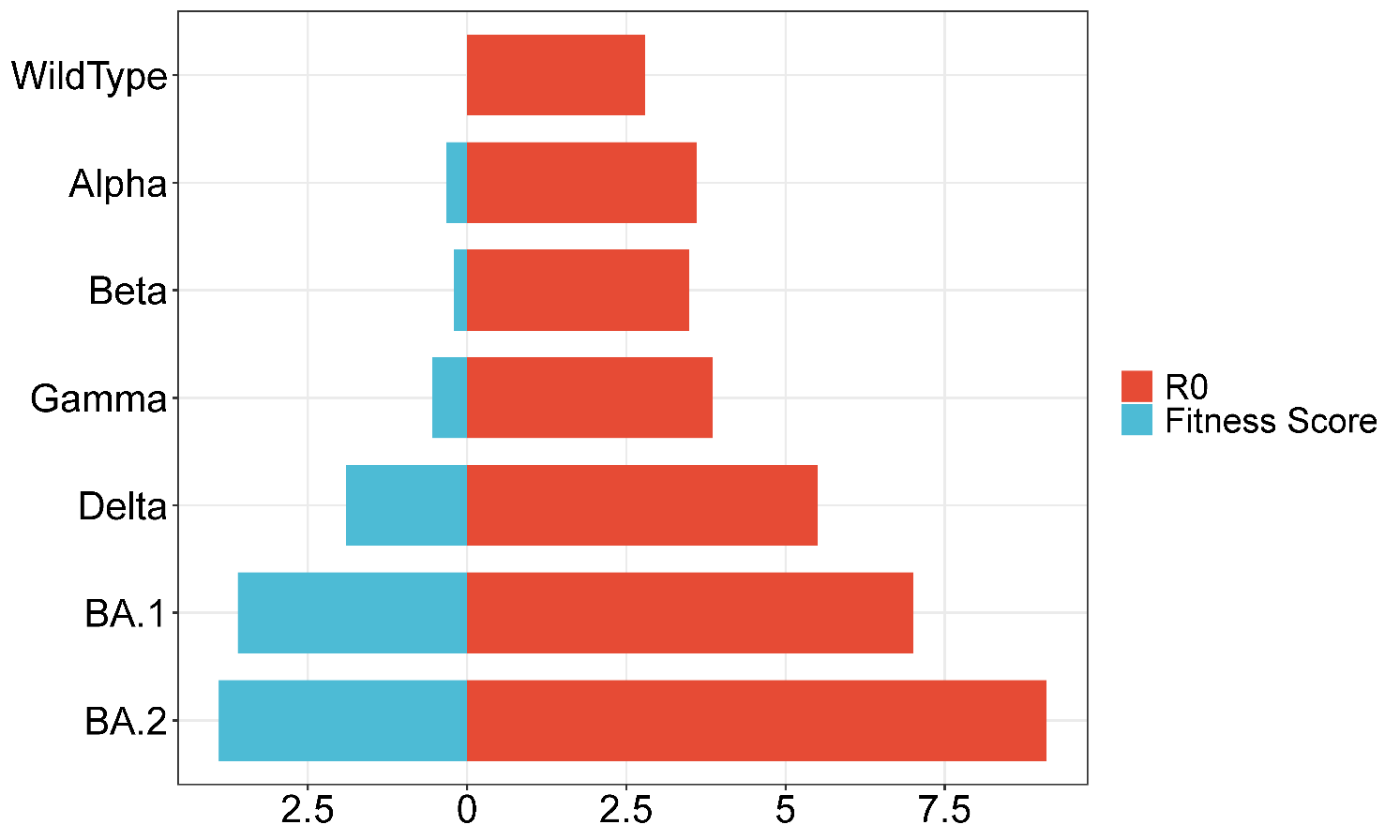


**Supplementary Figure 6.** Fitness score and R0 of wildtype and VoC strains. For Omicron strains, BA.1 and BA.2 are investigated. The fitness score of the wildtype is zero.


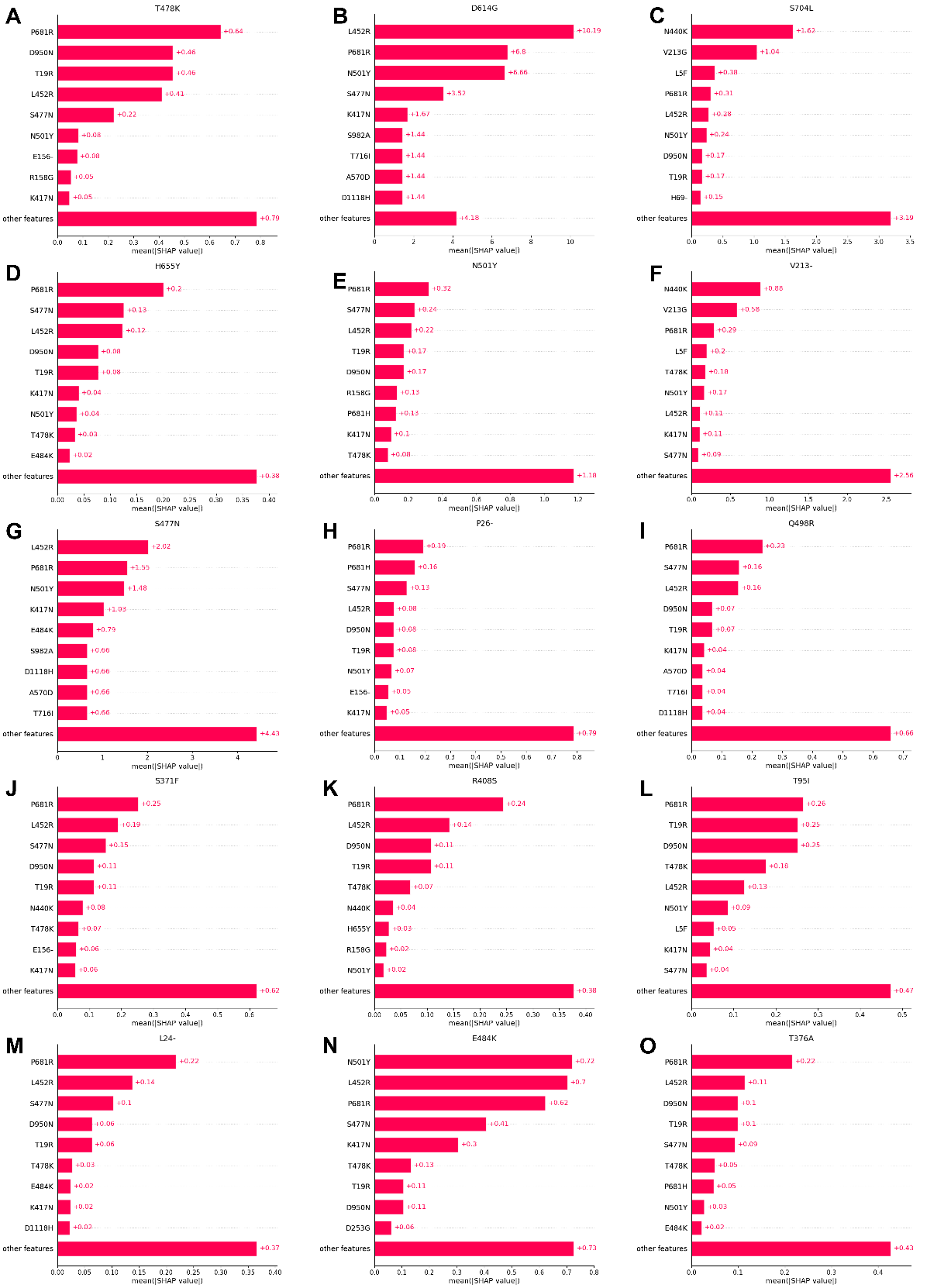


**Supplementary Figure 7.** Mean absolute value of SHAP values in the causal inference model for top mutations. **(A)** T478K. **(B)** D614G. **(C)** S704L. **(D)** H655Y. **(E)** N501Y. **(F)** V213-. **(G)** S477N. **(H)** P26-. **(I)** Q498R. **(J)** S371F. **(K)** R408S. **(L)** T95I. **(M)** L24-. **(N)** E484K. (**O)** T376A. Top features in the model are explicitly shown with values.


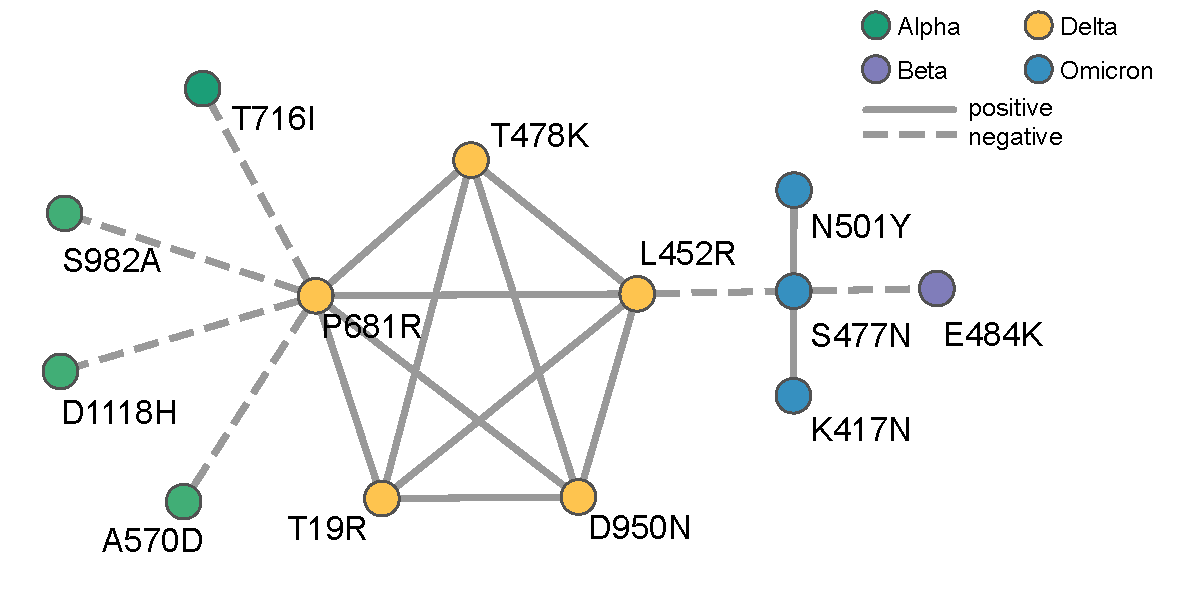


**Supplementary Figure 8.** Possible interactions between mutations, colored by related VoC variants. Positive and negative interactions are represented by different line types.

References

1. Bloom JD, Neher RA. Fitness effects of mutations to SARS-CoV-2 proteins. bioRxiv. 2023:2023.01.30.526314.

2. Cao Y, Wang J, Jian F, Xiao T, Song W, Yisimayi A, et al. Omicron escapes the majority of existing SARS-CoV-2 neutralizing antibodies. Nature. 2022;602(7898):657-63.

3. Kemp SA, Collier DA, Datir RP, Ferreira I, Gayed S, Jahun A, et al. SARS-CoV-2 evolution during treatment of chronic infection. Nature. 2021;592(7853):277-82.

4. Liu Y, Liu J, Johnson BA, Xia H, Ku Z, Schindewolf C, et al. Delta spike P681R mutation enhances SARS-CoV-2 fitness over Alpha variant. Cell reports. 2022;39(7):110829.

5. McCallum M, De Marco A, Lempp FA, Tortorici MA, Pinto D, Walls AC, et al. N-terminal domain antigenic mapping reveals a site of vulnerability for SARS-CoV-2. Cell. 2021;184(9):2332-47.e16.

6. Queirós-Reis L, Gomes da Silva P, Gonçalves J, Brancale A, Bassetto M, Mesquita JR. SARS-CoV-2 Virus-Host Interaction: Currently Available Structures and Implications of Variant Emergence on Infectivity and Immune Response. International journal of molecular sciences. 2021;22(19).

7. Zhang C, Wang Y, Zhu Y, Liu C, Gu C, Xu S, et al. Development and structural basis of a two-MAb cocktail for treating SARS-CoV-2 infections. Nature communications. 2021;12(1):264.

8. Stone JE, Vandivort KL, Schulten K, editors. GPU-accelerated molecular visualization on petascale supercomputing platforms. Proceedings of the 8th International Workshop on Ultrascale Visualization; 2013.

9. Humphrey W, Dalke A, Schulten K. VMD: visual molecular dynamics. Journal of molecular graphics. 1996;14(1):33-8, 27-8.
